# HMGB1 Promotes Intraoral Palatal Wound Healing through RAGE-Dependent Mechanisms

**DOI:** 10.3390/ijms17111961

**Published:** 2016-11-23

**Authors:** Salunya Tancharoen, Satoshi Gando, Shrestha Binita, Tomoka Nagasato, Kiyoshi Kikuchi, Yuko Nawa, Pornpen Dararat, Mika Yamamoto, Somphong Narkpinit, Ikuro Maruyama

**Affiliations:** 1 Department of Pharmacology, Faculty of Dentistry, Mahidol University, Bangkok 10400, Thailand; salunya.tan@mahidol.ac.th (S.T.); kikuchi_kiyoshi@kurume-u.ac.jp (K.K.); pornpen.dar@mahidol.ac.th (P.D.); 2 Department of Emergency and Critical Care, Hokkaido University, Kita-ku, Sapporo 0608648, Japan; gando@med.hokudai.ac.jp; 3 Department of Systems Biology in Thromboregulation, Kagoshima University Graduate School of Medical and Dental Science, Kagoshima 8908544, Japan; binita@m2.kufm.kagoshima-u.ac.jp (S.B.); n-tomoka@m.kufm.kagoshima-u.ac.jp (T.N.); m-yamamo@m2.kufm.kagoshima-u.ac.jp (M.Y.); 4 Division of Brain Science, Department of Physiology, Kurume University School of Medicine, Asahi-machi, Kurume 8300011, Japan; 5 Department of Anesthesiology and Intensive Care, Hokkaido Medical for Child Health and Rehabilitation, Sapporo 0060041, Japan; nawa@sapmed.ac.jp; 6 Department of Pathobiology, Faculty of Science, Mahidol University, Bangkok 10400, Thailand; somphong.nar@mahidol.ac.th

**Keywords:** HMGB1, NF-κB, wound healing, palatal mucosa

## Abstract

High mobility group box 1 (HMGB1) is tightly connected to the process of tissue organization upon tissue injury. Here we show that HMGB1 controls epithelium and connective tissue regeneration both in vivo and in vitro during palatal wound healing. Heterozygous HMGB1 (*Hmgb1*^+/−^) mice and Wild-type (WT) mice were subjected to palatal injury. Maxillary tissues were stained with Mallory Azan or immunostained with anti-HMGB1, anti-proliferating cell nuclear antigen (PCNA), anti-nuclear factor-κB (NF-κB) p50 and anti-vascular endothelial growth factor (VEGF) antibodies. Palatal gingival explants were cultured with recombinant HMGB1 (rHMGB1) co-treated with siRNA targeting receptor for advanced glycation end products (RAGEs) for cell migration and PCNA expression analysis. Measurement of the wound area showed differences between *Hmgb1*^+/−^ and WT mice on Day 3 after wounding. Mallory Azan staining showed densely packed of collagen fibers in WT mice, whereas in *Hmgb1*^+/−^ mice weave-like pattern of low density collagen bundles were present. At three and seven days post-surgery, PCNA, NF-κB p50 and VEGF positive keratinocytes of WT mice were greater than that of *Hmgb1*^+/−^ mice. Knockdown of RAGE prevents the effect of rHMGB1-induced cell migration and PCNA expression in gingival cell cultures. The data suggest that HMGB1/RAGE axis has crucial roles in palatal wound healing.

## 1. Introduction

In tissues skin and oral mucosa tissues, wound healing encompasses a number of overlapping phases, including inflammation, tissue formation and tissue remodeling [1]. The process of wound repair requires a complex and dynamic interplay of a number of tenant epithelial and mesenchymal cells with hematopoietic cells to accomplish the three stages of wound healing [2]. In the inflammation process, the primary phase is characterized by a local activation of innate immune mechanisms resulting in an initial influx of neutrophils into the injured tissue followed by macrophages accumulation. Subsequently, epithelialization proceeds gradually from the wound edges. Finally, tissue remodeling occurs during maturation of the newly formed tissues, which leads to scar formation [3]. Recently, wound healing analysis in a number of murine knockout models deficient for distinct inflammatory mediators including tumor necrosis factor (TNF)-α, interleukin (IL)-6, monocyte chemotactic protein (MCP)-1, and interferon (IFN)-γ have been investigated [4]. Dermal wound healing is accelerated in TNF-receptor-55 [5] or IFN-γ-deficient mice [6] but is impaired in mice deficient of IL-6 [7]. Likewise, palatal wound healing is accelerated in Smad3 [8] and interleukin (IL)-10 [9] deficient mice.

High-mobility group box 1 (HMGB1) is a ubiquitous 25 kDa nuclear DNA-binding protein that, under typical conditions, is located in the nucleus, where it organizes the basic structural unit of chromatin, DNA replication, and transcription. HMGB1 also acts in the extracellular environment as a primary pro-inflammatory cytokine [10]. Upon *Prevotella intermedia* lipopolysaccharide stimulation, odontoblast-like cells respond by upregulating receptor for advanced glycation end products (RAGEs) and producing HMGB1. In addition, HMGB1, a novel cytokine, is known to contribute to the pathogenesis of various inflammatory diseases [11,12,13], including periodontal diseases [14]. This suggests that HMGB1 can activate an inflammatory response during infection or injury. On the contrary, a role for HMGB1 in wound repair has been reported by the results of earlier studies. HMGB1 promotes neuronal differentiation of embryonic stem cells and neurite outgrowth in the developing nervous system [15]. Moreover, HMGB1 is a potent chemoattractant and mitogen for blood vessel-associated stem cells [16]. Limana et al. demonstrated that intracardiac HMGB1 injection in a mouse model of myocardial infarction induced new myocyte formation and improved infarcted hearts function [17]. In addition, HMGB1-induced increase of HaCaT keratinocytes proliferation, cell migration, and wound closure via RAGE and extracellular signal-regulated kinase (ERK) pathway [18]. RAGE is a member of the immunoglobulin superfamily and is expressed on gingival epithelial and fibroblast cells [19], mononuclear phagocytes, vascular smooth muscle cells, and neurons [20,21]. RAGE interacts with a range of ligands, including advanced glycation end products (AGEs), HMGB1, and S100/calgranulins [22,23]. Ligand binding results in RAGE-dependent sustained nuclear factor-kappa B (NF-κB) activation [24] as well as in wound healing promotion [25]. These reports indicate that HMGB1 is a multifunctional cytokine involved in inflammatory responses and tissue repair. Despite this, whether and how HMGB1 contributes to protective and/or pathological responses of palatal wound healing in vivo is unclear. In this study, we provide evidence that the loss of *Hmgb1* gene in HMGB1-heterozygous (*Hmgb1*^+/−^) mice results in delayed palatal wound closure. In addition, RAGE silencing reduces closure of an in vitro scratch wound gingival cell migration and proliferating cell nuclear antigen (PCNA) expression. Our results, show for the first time, that HMGB1/RAGE axis has crucial roles in palatal wound healing, by regulating collagen accumulation, cell proliferation and cell migration. Taken together, these results bring scientific support to the possible application of HMGB1 in regenerative medicine.

## 2. Results

### 2.1. Identification and Targeted Disruption of the Mouse Gene

**Figure 1:**
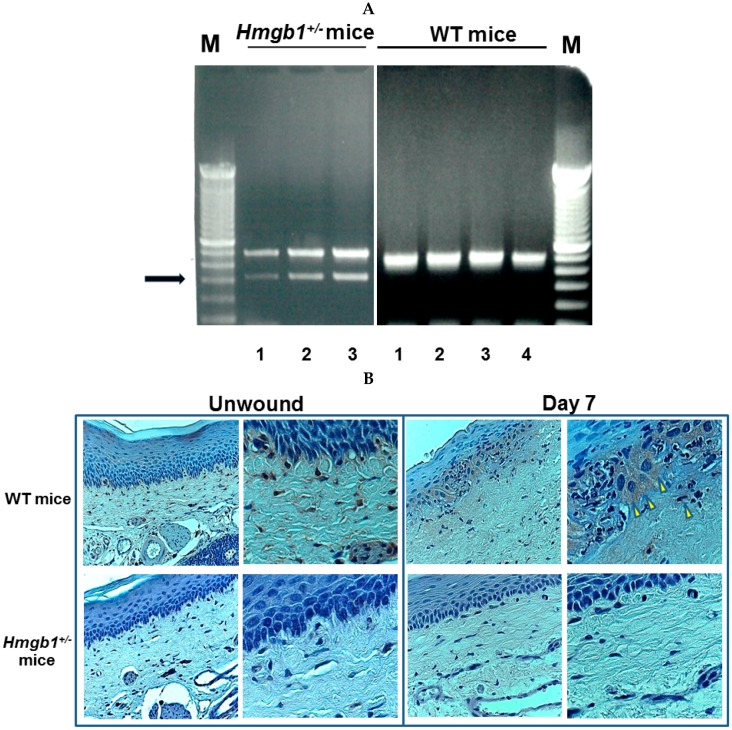
RT-PCR transcripts of mouse tail and immunohistochemistry determination of palatal section for *Hmgb1*^+/−^ and WT mice. (**A**) PCR products were separated in ethidium-bromide-stained agarose gels with DNA molecular weight marker (M, 100 bp ladder). PCR with the primer complementary to the gene identifies *Hmgb1*^+/−^ mice, shows two bands, a bright band at 500 bp (**upper**) and another at about 300 bp (**lower**). The predicted size of HMGB1 in WT and *Hmgb1*^+/−^ mice was 495 and 336 bp, respectively. Arrow indicates target allele of *Hmgb1*^+/−^ mice and (**B**) Immunohistochemical analysis of HMGB1 in palatal section of unwounded (Day 0) and wounded site (Day 7). Arrowheads indicate HMGB1 positive staining in the cytosol. Original magnifications: left panels 400×, right panels 800×. *n* = 3–5 for each group.

DNA extraction from mouse tail and genotyping PCR separated by agarose gel electrophoresis has been performed in *Hmgb1*^+/−^ and Wild-type (WT) mice (Figure 1A). PCR with the HMGB1 primer pair, for the mouse genome identifies WT mice, in which the product is detected at about 500 bp band (Figure 1A, lanes 1–4). On the other hand, PCR with the primer complementary to the gene identifies *Hmgb1*^+/−^ mice, shows a bright band at about 500 bp (amplification control) and another at about 300 bp (Figure 1A, lanes 1–3). The target allele of PCR product is 495 bp for WT mice and 336 bp for *Hmgb1*^+/−^ mice. Our data replicated previous findings that *Hmgb1*^+/−^ mice contained approximately one-half the level of HMGB1 mRNA compared with WT mice [26]. This decrease is confirmed by immunohistochemistry of HMGB1 protein within the unwounded site (Day 0) and experimental wound (Day 7) of hard palate mucosa in mice. By using a specific anti-HMGB1 polyclonal rabbit antibody (Ab) that does not detect HMGB2 and HMGB3 [27], we found that at seven days post-surgery, tissues from the WT mice presented higher expression of HMGB1 in the periphery of the nucleus together with some translocation from the nucleus to the cytoplasm of basal epithelial cells and cells of connective tissue compared with that in unwounded site (Figure 1B). Interestingly, marked differences in the intensity and localization of HMGB1 staining in unwounded and wounded sites were observed between *Hmgb1*^+/−^ and WT mice. Very faint staining of HMGB1 in *Hmgb1*^+/−^ mice tissue was shown and the pattern of HMGB1 expression was mainly intranucleus in basal keratinocytes. These results indicate that the positive staining of HMGB1 in hard palate mucosa of WT mice was diminished in *Hmgb1*^+/−^ mice.

### 2.2. Wound Closure Is Attenuated in Hmgb1^+/−^ Mice

**Figure 2:**
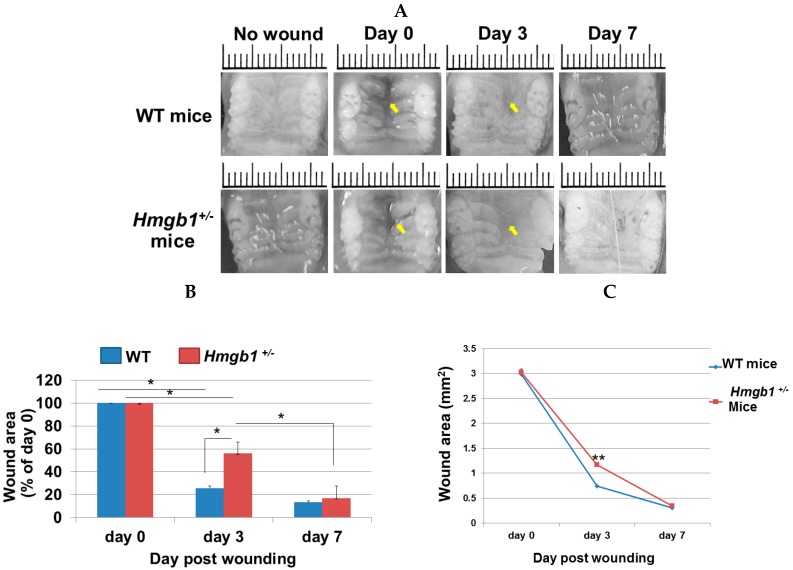
Wound closure is attenuated in *Hmgb1*^+/−^ mice vs. WT mice. (**A**) Macroscopic appearance of wounds of *Hmgb1*^+/−^ and WT mice at indicated time points after injury. Arrows indicate the wound bed or scar margins. Scale bars: 1.0 mm; (**B**) At the time points indicated, the wound area was analyzed and expressed as the percentage of the initial wound areas; (**C**) The reduction in wound area in WT and *Hmgb1*^+/−^ mice in mm^2^. Data are expressed as mean ± SD, *n* = 5 wounds for each time point and genotype (* *p* < 0.001, ** *p* < 0.05 vs. the WT group).

Macroscopic wound closure was attenuated in *Hmgb1*^+/−^ mice compared with WT animals. At three days post-surgery, it was observed that mucosal closure was not completed in the *Hmgb1^+/−^* mice (Figure 2A). The wound of WT mice appeared epithelialized, whereas the mutant wounds showed partial epithelialization. At seven days post-surgery, wound healing was more favorable in incision areas in the WT group than that of *Hmgb1*^+/−^ group. Measurement of the wound area on digital images showed that the differences between *Hmgb1^+/−^* (55.8% ± 1.48% of Day 0) and WT mice (25.6% ± 0.7% of Day 0) were statistically significant on Day 3 after wounding (*p* < 0.001, Figure 2B). Wound area assessment demonstrated significantly larger wounds in *Hmgb1^+/−^* mice compared to WT controls at Day 3 (1.2 ± 0.06 mm^2^ vs. 0.7 ± 0.04 mm^2^; *p* < 0.05) after wounding, whereas there was no statistically significant difference (*p* > 0.05) in the wound area between both groups at Day 7 (Figure 2C). The wound areas on Days 0, 3, and 7 were measured by three examiners. Pearson’s correlation coefficient (*r*) was used to show the correlation between the examiners (Figure S1). In WT mice, we found a statistically significant correlation between examiner 1 and examiner 2 (*r* = 0.9992, *p* < 0.001); examiner 1 and examiner 3 (*r* = 0.9992, *p* < 0.001); and examiner 2 and examiner 3 (*r* = 0.9998, *p* < 0.001). Likewise, we demonstrated a statistically significant correlation between examiner 1 and examiner 2 (*r* = 0.9909, *p* < 0.05); examiner 1 and examiner 3 (*r* = 0.9902, *p* < 0.01); and examiner 2 and examiner 3 (*r* = 0.9906, *p* < 0.01) in *Hmgb1*^+/−^ mice. According to the macroscopic results, we concluded that *Hmgb1*^+/−^ mice showed a significant delay in wound healing as compared to WT mice (seven days vs. three days).

### 2.3. Reduction of Collagen Fibers and Delayed Re-Epithelialization in HMGB1^+/−^ Wound

**Figure 3:**
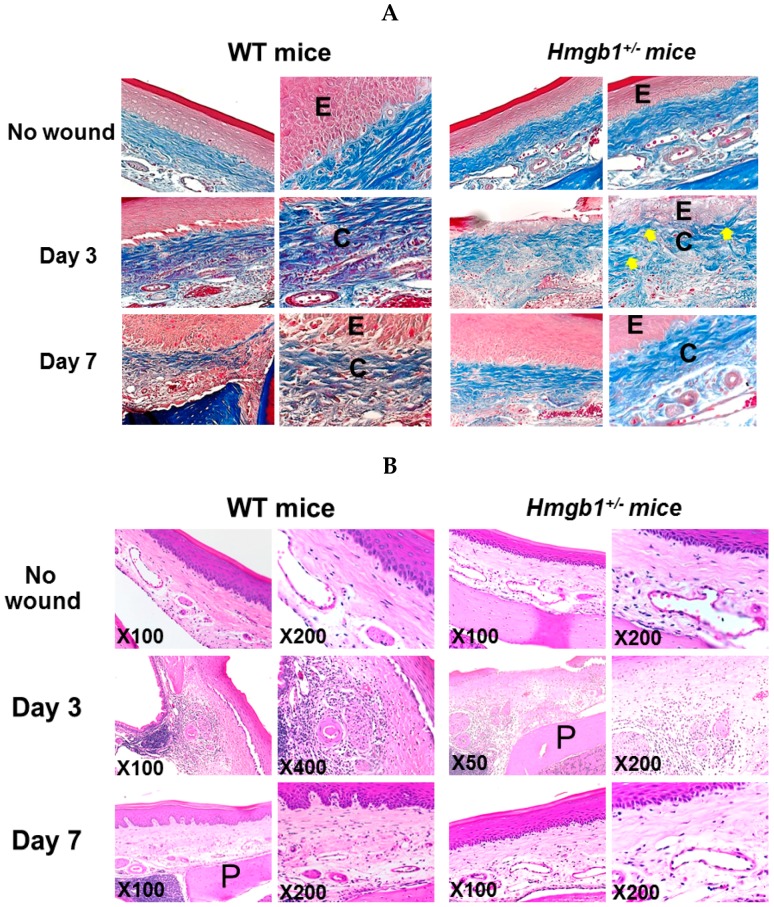
Reduction of collagen fibers and delayed re-epithelialization in *Hmgb1*^+/−^ wound on Day 3 after wound surgery. (**A**) Sections were stained with Mallory’s azan. Representative palatal wound sections show the collagen bundles of WT and *Hmgb1*^+/−^ mice. Note that blue indicates collagen bundle stained by Mallory’s azan stain. Arrows indicate weave-like pattern of collagen bundles in *Hmgb1^+/−^* mice. Original magnifications: left panels 200×, right panels 400×; (**B**) Sections were stained with hematoxylin and eosin. E, epithelium; C, collagen bundle; P, palatal bone. *n* = 5 wounds for each time point and genotype.

Collagen components are a major part of oral mucosa development [28]. The macroscopic findings of wound closure were confirmed by histological assessment. At three days post-surgery, delayed wound healing was determined in the *Hmgb1*^+/−^ group compared to the WT group. Mallory Azan staining of Day 3 wound showed well-organized, parallel, densely packed and thick bundles of collagen fibers in WT mice, whereas in *Hmgb1*^+/−^ mice weave-like pattern of low density collagen bundles were present (Figure 3A). At seven days post-surgery, the collagen fibers were prominently mature and re-epithelialization was observed in wound regions of both groups. A corresponding hematoxylin and eosin (H & E)-stained wound section is shown in Figure 3B for comparison. In the *Hmgb1*^+/−^ group, subepithelial healing was evidenced but was not completed and, in connective tissue region, mononuclear cell infiltration was present at three days post-surgery. More prominent infiltration of mononuclear cell infiltration was present in WT mice than in *Hmgb1*^+/−^ mice. At Day 7, the wound healing properties were not different in *Hmgb1*^+/−^ and WT mice. Collectively, these findings show significant delays in wound healing parameters, including epithelialization and decreased collagen formation in *Hmgb1*^+/−^ mice.

### 2.4. Immunohistochemistry Determination of Proliferating Cells at Palatal Wounds in WT and Hmgb1^+/−^ Mice

**Figure 4:**
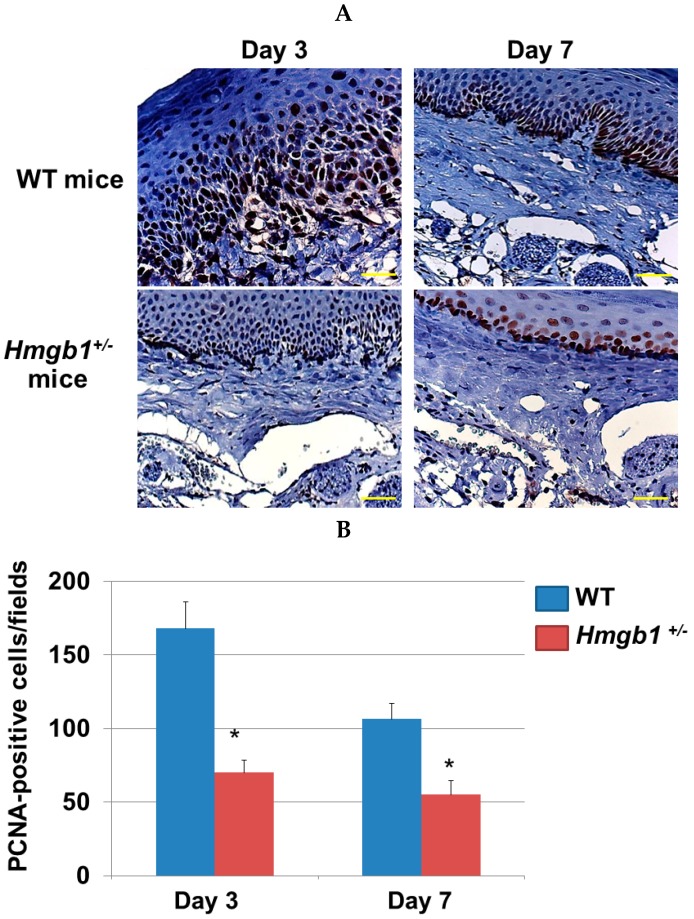
Distribution of proliferating cells at palatal wounds in WT and *Hmgb1*^+/−^ mice. (**A**) Analysis of cell proliferation by immunohistochemistry stained with the anti-PCNA Ab in the palatal wounds on Day 3 and Day 7 post surgery. Scale bars = 100 μm; (**B**) The number of PCNA-positive cells per field in palatal wound. Data are expressed as the mean ± SD. (*n* = 5–8 for each group). * *p* < 0.001 vs. the WT group.

To identify the mechanism underlying the attenuated palatal wound closure in *Hmgb1*^+/−^ mice, we assessed cell proliferation in the repaired tissue by immunohistochemistry, using PCNA Ab. PCNA is expressed in both basal and suprabasal cell layers (Figure 4A). At three days post-surgery, the numbers of PCNA-positive keratinocytes in WT mice wound site (168 ± 18) were significantly greater than *Hmgb1*^+/−^ mice (70 ± 8.7) (Figure 4B, *p* < 0.001). At seven days post-surgery, PCNA-positive keratinocytes numbers are reduced in both groups. The values were significantly higher (*p* < 0.001) in WT mice (106.5 ± 10.4) than *Hmgb1*^+/−^ mice (55 ± 9.7). From the above results, we conclude that the proliferation marker, PCNA, was significantly lower in the *Hmgb1*^+/−^ mice compared with WT mice.

### 2.5. Localization of NF-κB p50 Isoform at Palatal Wounds in WT and Hmgb1^+/−^ Mice

**Figure 5:**
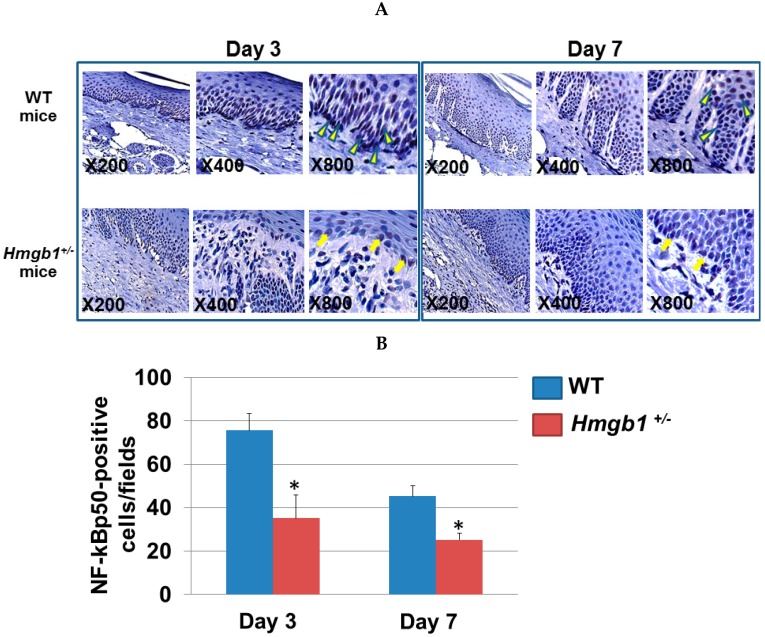
Localization of NF-κB p50 isoform at palatal wounds in WT and *Hmgb1*^+/−^ mice. (**A**) By immunohistochemistry, slides were stained with the anti-NF-κB p50 Ab in the palatal wounds on Day 3 and Day 7 post surgery. Nuclei were counterstained with Mayer’s hematoxylin. Arrowheads indicate NF-κB p50-positive stained nuclei of epithelial cells. Arrows indicate faint immunostaining of NF-κB p50 in *Hmgb1*^+/−^ mice; (**B**) The number of NF-κB p50-positive cells per field in palatal wound. Data are expressed as the mean ± SD. Error bars indicate standard deviation (*n* = 5 for each group). * *p* < 0.05 vs. the WT group.

Blocking HMGB1 can decrease the degree of radiation-induced pulmonary damage, and its mechanism may be related to the promotion of NF-κB p50 activation and its downstream molecular expression [29]. NF-κB p50 antigen in epithelial cells was examined in serial sections of the same tissue block (Figure 5A). At three days post-surgery, NF-κB p50-immunopositive cells in WT mice wound site (75.6 ± 7.8) were significantly greater (*p* < 0.05) than *Hmgb1^+/−^* mice (35.1 ± 4.9). At seven days post-surgery, NF-κB p50-positive cell numbers are reduced in both groups. The values were significantly higher (*p* < 0.05) in WT mice (45.1 ± 10.5) than *Hmgb1^+/−^* mice (25.1 ± 2.9) (Figure 5B). These results indicated that pro-inflammatory signaling pathway, NF-κB was significantly lower in the *Hmgb1^+/−^* mice compared with WT mice. No tissues in samples from WT and *Hmgb1^+/−^* group were stained with isotype-matched control IgG (Figure S2).

### 2.6. Determination of VEGF Expression and Localization in Palatal Wounds of WT and Hmgb1^+/−^ Mice

**Figure 6:**
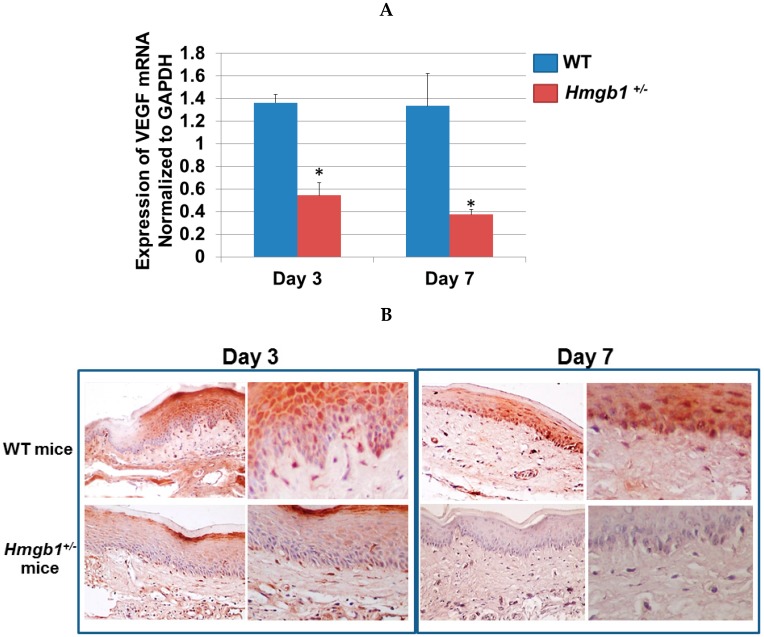
Determination of VEGF mRNA expression and VEGF protein immunolocalization in the palatal wounds of WT and *Hmgb1*^+/−^ mice. (**A**) Expression of VEGF mRNA in palatal wounds on Day 3 and Day 7 post surgery was quantified by real time PCR analysis and reported by normalized to GAPDH. Data are expressed as the mean ± SD of at least three independent determinations. * *p* < 0.001 vs. the WT group; (**B**) Analysis of angiogenic response by immunohistochemistry stained with the anti-VEGF Ab. Original magnifications: left panels 400×, right panels 800×. Experiments were performed in triplicate.

The expression of VEGF within the area of granulation tissue was used as read-out for neovascular processes [30]. Real time PCR analysis demonstrated the ratio of VEGF normalized to the glyceraldehyde-3-phosphate dehydrogenase (GAPDH) transcript content in palatal wound site of *Hmgb1^+/−^* and WT mice (Figure 6A). At three days post-surgery, VEGF mRNA in WT mice wound site (1.4 ± 0.07) were significantly greater (*p* < 0.001) than *Hmgb1^+/−^* mice (0.5 ± 0.1). There was no statistically significant difference of VEGF values between three days and seven days post-surgery in WT mice (*p* > 0.05). At seven days post-surgery, the VEGF value was significantly higher (*p* < 0.001) in WT mice (1.3 ± 0.3) than *Hmgb1^+/−^* mice (0.37 ± 0.05). Next, we examined by immunohistochemistry the presence of VEGF protein within the wound site at three and seven days after surgery. At three days post-surgery, VEGF was detected in the oral epithelia above the basal layer (Figure 6B) to the spindle cell layers with rising density in the wound site of WT mice. In *Hmgb1^+/−^* group, on the other hand, specific labeling was faint or negative, indicating that VEGF is poorly expressed in these cells. There was also scattered VEGF in capillary endothelial cells, infiltrating inflammatory cells, and fibroblast-like cells in the connective tissue of all WT mice and *Hmgb1^+/−^* mice wound sites. At seven days post-surgery, VEGF was detected in the whole layer of oral epithelia; whereas in the *Hmgb1^+/−^* group, VEGF is absent in these cells. No tissues in samples from WT and *Hmgb1^+/−^* group were stained with isotype-matched control IgG (Figure S2). Collectively, these results demonstrate that HMGB1 ablation contributes to the delayed angiogenic response in heterozygous mouse model.

### 2.7. Efficiency of RAGE Gene Knockdown

**Figure 7:**
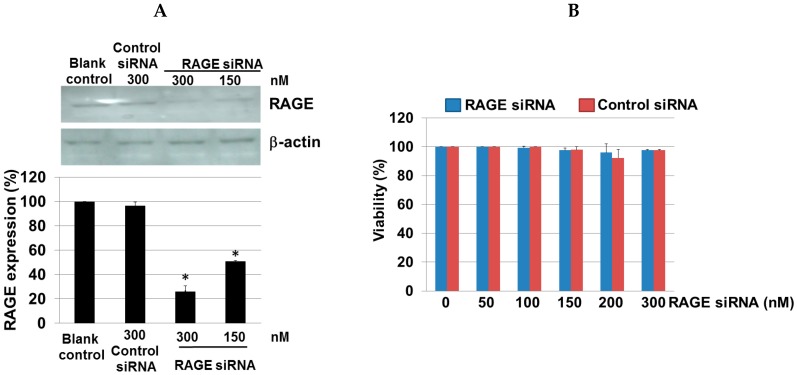
Efficiency of RAGE gene knockdown in gingival epithelial cells. (**A**) A representative Western blot using antibodies against RAGE and β-actin is shown. The results showed decreased expression of RAGE protein in the siRNA group compared to the control siRNA and blank control groups. The intensity of the protein bands in Western blotting was quantified by densitometry and normalized to β-actin. Three independent measurements were performed. Data are expressed as mean ± SD. * *p* < 0.001 vs. the control group; (**B**) Cytotoxicity of the siRNA complexes was assessed by MTT assay. There was no evidence of cell toxicity found in the RAGE siRNA transfected cells. Data are expressed as the mean ± SD. Error bars indicate SD of at least three independent determinations.

The silencing of RAGE gene expression was carried out following the transfection of RAGE-specific siRNA in gingival epithelial cells (Figure 7A). Western blot analysis showed that both 150 and 300 nM of RAGE siRNA significantly decreased the expression of RAGE protein by 50% and 70%, respectively, without affecting house-keeping gene (β-actin) expression or any toxicities after transfection (MTT assay; Figure 7B).

### 2.8. RAGE Silencing Reduces Closure of an In Vitro Scratch Wound and PCNA Expression

**Figure 8:**
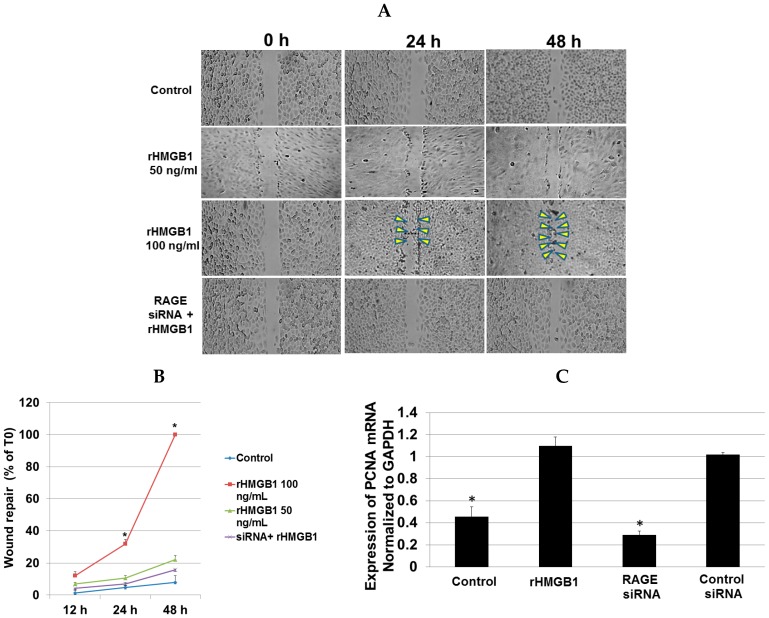
RAGE silencing reduces closure of an in vitro scratch wound, gingival epithelial cell migration and PCNA expression. (**A**) Representative photomicrographs of a cell migration assay. Cells were pretreated with 300 nM siRNA against RAGE and stimulated with rHMGB1 at different concentrations in culture media containing 2% FBS for the cell migration assay with 20× magnification. Treatment with rHMGB1 (100 ng/mL) increases the ability of cells to migrate into the empty area and to repair the wound at all-time points (24 and 48 h) examined. Yellow arrows indicate migration into the cell free-space; (**B**) The ability of cells to migrate and cover the empty space was determined as a wound repair by percent of 0 h (T0). Data are mean ± SD of 3–5 independent experiments. * *p* < 0.001 vs. the control siRNA and blank control groups; (**C**) Expression of PCNA mRNA in the cells after addition of 300 nM RAGE siRNA or control siRNA and stimulation by 100 ng/mL rHMGB1 was quantified by real time PCR analysis and reported by normalized to GAPDH. Data are expressed as the mean ± SD of three independent determinations. * *p* < 0.001 vs. the rHMGB1 group.

A cell monolayer scratch assay is used to evaluate keratinocyte proliferation and migration during re-epithelialization [31]. To assess whether delayed in the wound healing at the wound sites of the *Hmgb1^+/−^* mice is accompanied by retarded re-epithelialization mechanism, gingival epithelial cells were subjected to wound-healing (scratch) assay, in which the ability of cells to migrate and cover the cell-free space is monitored. Effects of recombinant HMGB1 (rHMGB1) on the cell migration were studied. Cells were either exposed to rHMGB1 at the concentration of 50 and 100 ng/mL or pretreated with RAGE siRNA. As shown in Figure 8A,B, untreated cells tend to close the wound by about 8% within 48 h. Interestingly, treatment with 100 ng/mL rHMGB1 enhanced cell migration by 32% and 100% at 24 and 48 h, respectively. Previous study reported that HMGB1 binds RAGE and promotes NF-κB activation [32]. In the present study, transfection of gingival epithelial cells with RAGE siRNA suppressed rHMGB1-induced cell migration by ~85% compared with cells treated with 100 ng/mL rHMGB1 for 48 h (Figure 8B). PCNA is considered to be a marker of cell proliferation [33]. In our study, immunohistochemical staining for PCNA in the wound section revealed that PCNA expression was significantly lower in the *Hmgb1^+/−^* mice compared with WT mice. We therefore sought to determine whether retarded in the wound healing of *Hmgb1^+/−^* mice is related to cell proliferation mechanism. Cells were treated with rHMGB1 at the concentration 100 ng/mL or pretreated with 300 nM RAGE siRNA (Figure 8C). Transcript levels of PCNA were significantly higher in the rHMGB1-treated cell than in the control group (*p* < 0.001). Knockdown of RAGE prevents the effect of HMGB1-induced PCNA expression in gingival epithelial cells by ~70% compared with cells treated with rHMGB1. No effects were observed on control siRNA-treated cells. These results thus suggested that knockdown of RAGE prevents the effect of HMGB1-induced cell migration and cell proliferation in gingival epithelial cells. In addition, HMGB1 promotes molecular signaling, leading to cell migration and proliferation via RAGE.

## 3. Discussion

The present study is, as far as we know, the first in which the regulatory role of HMGB1 and its potential involved signal pathways in palatal wound healing has been examined. We show that wound healing is attenuated in *Hmgb1*^+/−^ mice compared with WT mice. In vitro studies demonstrate cell mobility and cell proliferation in gingival epithelial cells after rHMGB1 stimulation. HMGB1 protein is a multifunctional cytokine involved in tissue inflammation and regeneration. Recent studies have documented an important role of HMGB1 in mediating tissue repair [15,16,17]. Straino et al. has shown that HMGB1 administration promotes wound healing in diabetic mice [26]. Furthermore, HMGB1 had a chemotactic effect on skin fibroblasts and keratinoyctes during wound healing process [18]. Additionally, HMGB1 was able to significantly induce proliferation of human gingival fibroblast and induce cell migration [27]. With these outcomes, it is interesting that HMGB1 may be important for wound healing and promote evidences which support our study.

Although the distribution of HMGB1 in relation to wound healing has been observed in human skin [26] and cholesteatoma [28], studies dealing with palatal wound healing and the target mutation of *Hmgb1* gene in vivo have not been previously performed. In our study, we found that HMGB1 expression in unwounded and wounded site of *Hmgb1*^+/−^ mice is very low compared to WT mice. In addition, a delayed epithelialization contributed to the attenuation of *Hmgb1*^+/−^ mice wound closure and was significantly difference from WT mice at three days post-surgery. These results imply that HMGB1 is responsible for palatal wound repair. HMGB1 is translocalized and released after inflammatory stimuli [29,30] and is mobilized from gingival epithelial cells in response to periodontal inflammation [31]. Our findings demonstrate that at seven days after surgery, tissues from the WT mice presented HMGB1 protein translocation from the nucleus to the cytoplasm of basal epithelial cells and cells of connective tissue, which is in accordance with results obtained in other cell types [29,30,31].

Palatal wound healing involves a complex, well-orchestrated series of events like hemostasis, inflammatory response and collagen synthesis. A previous study has demonstrated that treatment with 100 ng/mL has a chemotactic effect on fibroblasts and keratinocytes. Following a single dose of 200 and 400 ng HMGB1, wound closure was accelerated in diabetes mice compared to wounds treated either with saline or 800 ng of this protein [26]. In our in vitro study, rHMGB1 at a concentration of 100 ng/mL induces gingival epithelial cells migration and PCNA mRNA overexpression. Accordingly, it is likely that HMGB1 promotes palatal wound healing by accelerating the re-epithelialization and proliferation of gingival epithelial cells, may occur in vivo. Nevertheless, another study showed that treatment with 100 ng/mL of HMGB1 protein impairs fibroblast collagen synthesis in rats undergoing full-thickness incisional wound. Reduction of HMGB1 levels in the wound using ethyl pyruvate leads to significant increases in reparative collagen deposition [32]. The difference in the results of these studies may be associated with the variations in fibroblast subpopulations and the proliferative potential of the cells in the lesion.

VEGF appears to play a crucial role in the proliferative phase of wound healing, promote migration, differentiation and tube formation of endothelial cells which are key elements in early stages of angiogenesis [33]. Our study demonstrated new vessels appear as early as three days after wounding and staining for VEGF was most prominent in keratinocytes at seven days post-surgery, consistent with VEGF expression results of previous reports [34,35,36]. HMGB1 administration restored the blood flow recovery in the ischemic muscle of diabetic mice is associated with the increased expression of VEGF [37]. In our study, we show that decreased VEGF expression in keratinocytes is associated with impaired wound healing in the *Hmgb1*^+/−^ mice. One explanation for this result is that about three to ten days after the wound occurs, macrophages are abundant in the wound tissue and new blood vessels are formed [38]. Activated macrophages and monocytes secrete HMGB1 [39] and this HMGB1 may in turn induce VEGF expression in keratinocytes. However, wound healing is a complex organized process including a variety of cell types (keratinocytes, endothelial cells, fibroblasts, inflammatory cells and epidermal cells) and angiogenesis inducers other than VEGF. Thus, despite an impairment of wound healing in *Hmgb1*^+/−^ mice in this study, further studies are required to rule out crucial biological processes involved in the early and late phase of wound repair in this model.

A cell monolayer scratch assay is used to assess re-epithelialization [40]. In the present study, we demonstrated a large induction of cell migration in gingival epithelial cells by rHMGB1 during wound scratch assay after 24–48 h. This finding is consistent with previous report that HMGB1 is a potent cell migration and proliferation promoting agent [41]. Nevertheless, HMGB1-induced increase in human gingival and periodontal ligament fibroblast migration after 16 h stimulation in the transwell chamber assay [27]. It is possible that the difference in the speed of cell migration in our experiment compared to the previous study may depend in part on particular cell type and assay protocol.

HMGB1 binding to RAGE triggers transcription factor NF-κB [42]. Knockdown of HMGB1 or RAGE inhibited NF-κB p50 and p65 expression [43]. Our study demonstrated that NF-κB p50-positive epithelial cells of WT mice were significantly higher than in *Hmgb1*^+/−^ mice. This data correlated with previous studies in which reduction in HMGB1-RAGE [43] or Toll-like receptor [44] axis reduced NF-κB activation and could not promote cellular proliferation. To elucidate the molecular mechanisms of HMGB1-induced wound re-epithelialization in vivo, we performed in vitro cell migration assay and PCNA transcription in gingival epithelial cells-treated with rHMGB1 in the presence or absence of RAGE siRNA. The result of our study demonstrates that HMGB1 signaling promotes wound healing of palatal mucosa via RAGE-dependent. It is noteworthy that intraoral wound healing process involves interaction of other signal molecules such as Smad3 [8] and transforming growth factor beta 1 [45]. Hence, the HMGB1 mouse model in our study may partly explain one of the biological factors that regulate palatal tissue repair and support the view that the local inflammatory response can promote wound closure. At the molecular level, these observations may be explained, at least in part, by the capacity of HMGB1 molecules in keratinocytes on palatal wound repair. As such, further analysis of specific effects of this protein may lead to novel therapies for improved wound healing properties.

Attempts have been made to repair and restore destroyed periodontal tissues, including the use of particular bioactive substances or surgical bone grafts [20,34]. Nowadays, conventional therapies are poorly effective on complex chronic ulcers and tissue lesions occurring in specific diseases, like diabetes. Topical application of HMGB1 to the wounds of streptozotocin-induced diabetic models in mice, enhanced arteriole density, granulation tissue formation, and accelerated wound healing in mice skin [26]. HMGB1-induced human gingival fibroblasts [27] and gingival epithelial cells proliferation and migration. These data suggest that HMGB1 may have potential beneficial effects on tissue remodeling and repair. Therefore, there is a great interest for a new generation of topical chronic wound treatments containing low levels of HMGB1 to accelerate intraoral wound healing and reduce wound-related complications.

## 4. Materials and Methods

### 4.1. Reagents

VEGF and HMGB1 Ab were obtained from Abcam (Cambridge, MA, USA). NF-κB p50 and PCNA Ab were purchased from Santa Cruz Biotechnology (Santa Cruz, CA, USA). Unless otherwise stated, all other reagents were supplied by Sigma-Aldrich Inc. (St. Louis, MO, USA).

### 4.2. Mice

All animal experiments were performed according to protocols approved by the institutional guidelines at Kagoshima University Graduate School of Medical and Dental Science. (Ethic approval number: H26/078, Approval date: 24 October 2014) Generation of Hmgb1 heterozygotes (*Hmgb1*^+/−^) mice on a pure BALB/c (Wild-type, WT) genetic background used in the present study were described before [46]. All mice were maintained and bred under standard pathogen-free conditions. Northern blot analysis of total RNA extracted from *Hmgb1*^+/−^ mice newborn liver using cDNA-encoding HMGB1 demonstrated approximately one-half the level of HMGB1 mRNA and protein compared with WT mice [46].

### 4.3. Polymerase Chain Reaction (PCR) Genotyping Assay

For genotyping PCR analysis, 3 mm sections of tail tip were dissolved in 0.1 mL of 50 mM Tris (pH 8.0), 100 mM EDTA, 0.5% SDS, and 0.5 mg/mL proteinase K solution at 55 °C for 2 to 6 h with vigorous shaking. DNA was prepared using DNeasy Blood Tissue Kit (Qiagen, Redwood City, CA, USA) and subjected to reverse transcription (RT)-PCR. We routinely genotyped newborn mice RT-PCR analysis of tail DNA (100 ng) using a commercial RT-PCR kit (Takara Biomedicals, Shiga, Japan), and the reaction was performed following the manufacturer’s instructions. The resulting cDNA mixture was amplified with Taq polymerase, and the following specific primers (Sigma, St. Louis, MO, USA) were used: WT allele, 5′-GCAGGCTTCGTTGTTTTCATACAG-3′ and 5′-TCAAAGAGTAATACTGCCACCTTC-3′, which generate a 495-bp fragment. The mutant *Hmgb1* allele was detected by using primer complementary to the neomycin resistance gene, 5′-TGGTTTGCAGTGTTCTGCCTAGC-3′ and 5′-CCCAGTCATAGCCGAATAGCC-3′ which generate a 336-bp fragment [11]. Amplification conditions were 1 cycle of 95 °C for 5 min, 35 cycles of 95 °C for 45 s, 60 °C for 30 s, 72 °C for 30 s with primer extension time at 72 °C for 5 min.

### 4.4. Palatal Wound Healing Model and Histological Analysis

The criteria used for assessing wound healing included; the epithelialization (as assessed by macroscopic evaluation for wound closure) [47], the degree of inflammation in the tissues [22] and collagen formation, which is responsible for tissue repair [48]. The macroscopic finding of continuous wound closure was confirmed by histology (H & E stain) and Mallory Azan. We determined the expression of cell proliferation (PCNA) and angiogenesis (VEGF) in wound tissue of the mice studied by immunohistochemistry. Eight- to fifteen-week-old mice were used in this study (5–7 mice per group). All animals were anaesthetized with isoflurane. A full-thickness incision wound, 1.0-mm width and 2.0-mm length, was made in the mucoperiosteum of the hard palate under sterile condition based on previous study with a slight modification [49]. After the incision was made, animals were sacrificed on Days 0, 3 and 7. No medication was used throughout the experiment. At the time points indicated, the wound kinetics were determined by using image-processing software (ImageJ, US National Institutes of Health, Bethesda, MD, USA) to measure the wound area; wound area was demonstrated as a percentage of the initial wound area. The wound closure was considered complete when the entire surface area was covered with tissue. The measurements were performed by three different examiners and blinded to each other. Pearson’s correlation coefficient was used to represent inter-examiner reliability.

To assess the wound healing process by microscopic examination, maxillary tissues after wounding were harvested. Tissues were fixed in 4% freshly made paraformaldehyde in 100 mM sodium phosphate buffer (pH 7.0) overnight at 4 °C. The samples were then decalcified with 19% EDTA in 100 mM PBS for 3 weeks, dehydrated, and embedded in paraffin. Serial sections, 4 μm thick, were cut in the frontal plane through the midpoints of the bilateral second molars. Slides were stained with H&E or Mallory Azan, and evaluated for the histological changes under light microscope (Jenamed II, Carl Zeiss, Gottingen, Germany).

### 4.5. Immunohistochemistry

Paraffin-embedded sections were deparaffinized in xylene and rehydrated through a series of decreasing concentrations of ethanol. Staining was carried out using indirect immunoperoxidase diaminobenzidine (DAB). Endogenous peroxidase was blocked by 0.3% H_2_O_2_ for 5 min. Sections were incubated for 1 h at room temperature with polyclonal anti-PCNA (1:200), NF-κB p50 (1:500), HMGB1 (1:200) and VEGF Ab (1:200) in Ab diluent with background reducing components (DakoCytomation, Carpinteria, CA, USA). As a negative control, the IgG isotype control was employed at the same time and concentration as the test antibodies. After rinsing with PBS, sections were finally developed with a DAKO LSAB+ System, horseradish peroxidase (HRP) (DakoCytomation; KO679), and immunostaining was visualized with substrate solution (DAB). Counterstaining was performed with Mayer’s hematoxylin. Immunostaining of PCNA and NF-κB p50 were localized in the nucleus of epithelial cells. VEGF staining was noticed in the cytoplasm. The number of PCNA and NF-κB p50 immunopositive cells from six slides in experimental and control samples were selected randomly and evaluated by two blinded observers and scored.

### 4.6. Quantitative Real-Time PCR Analysis of VEGF Expression in the Wound Tissues

Total RNA was extracted from palatal wound tissues of WT mice or *Hmgb1*^+/−^ mice using TRIzol Reagent (Invitrogen, Carlsbad, CA, USA). Total RNA samples (2 μg) were reverse transcribed using a First Strand complementary DNA synthesis kit for RT-PCR (Roche, Indianapolis, IN, USA). cDNA was augmented by real-time RT-PCR (*C*_t_ value 20–30 s cycles) using a 7300 Real-Time PCR System (Applied Biosystems, Foster City, CA, USA). The primers for gene amplification were as follows: VEGF sense, 5’-AACGATGAAGCCCTGGAGTG-3’; VEGF antisense, 5’-GACAAACAAATGCTTTCTCCG-3’ (accession number MN_00441242); GAPDH sense, 5’-TGTGTCCGTCGTGGATCTGA-3’; GAPDH antisense, 5’-CCTGCTTCACCACCTTCTTGAT-3’ (accession number NM_008084.3). The PCR conditions were 95 °C for 2 min followed by 40 cycles of 95 °C for 15 s, annealing at 58 °C for 30 s, and extension at 72 °C for 15 s. All reactions were run in triplicate. VEGF expression was defined on the basis of the threshold cycle (*C*_t_ value) and normalized to the GAPDH expression.

### 4.7. Primary Cell Cultures

Palatal gingival explants were prepared for the cell culture according to our previous study [50] with some modification. Briefly, the gingival tissues were surgically removed from the animal, placed in tissue culture plates and soaked in Dulbecco’s-modified Eagle Medium (DMEM; Sigma-Aldrich, St. Louis, MO, USA) containing 10% fetal bovine serum (FBS). After 2 weeks, gingival epithelial cells were harvested from the culture medium and further incubated in Keratinocyte-Serum Free Medium (Life Technologies, Rockville, MD, USA) supplemented with epidermal growth factor (5 ng/mL) and bovine pituitary extract (30–50 μg/mL). The cells of passages 4–6 were used for experiments. All cells were cultured in serum-free Opti-MEM-I medium (Gibco, Grand Island, NY, USA) for at least 15 h before treatment to eliminate the possible side effect of growth factors.

### 4.8. Silencing of RAGE Gene Expression and Western Blot Detection for RAGE Protein Expression

RNA silencing was performed with siRNA targeting RAGE mRNA or control siRNA (Santa Cruz, catalog: sc-36375) prepared according to the method described in our previous study [29]. Cells were transfected with siRNA duplexes suspended in lipofectamine reagent (Life Technologies) following the manufacturer’s protocol. Briefly, cells were cultured in 6-well plates until 60% confluence. Cells were washed with serum-free Opti-MEM I Reduced Serum Medium (Gibco, Grand Island, NY, USA). RAGE siRNA and negative control siRNA were gently premixed with lipofectamine reagent in Opti-MEM medium for 20 min at RT. The siRNA (final concentration 150–300 nM)/lipofectamine reagent complex was overlaid onto the washed cells for an additional 24 h at 37 °C in 5% CO_2_. The efficacy of gene silencing was evaluated using Western blot analysis. Protein concentrations were determined by Bradford protein assay using bovine serum albumin as standard (Bio-Rad, Hercules, CA, USA). Samples were mixed with 2× electrophoresis sample buffer solution with bromophenol blue (Santa Cruz Biotechnology) before being subjected to 12% SDS-polyacrylamide gel electrophoresis (PAGE) and transferred onto nitrocellulose membranes (Schleicher & Schuell, Dassel, Germany). Samples containing 10 or 15 μg of total protein were used. To prevent nonspecific binding, the membrane was blocked with a solution containing 5% (*w*/*v*) nonfat dry milk with 1% (*v*/*v*) Tween 20 in PBS for 1 h at RT. Rabbit anti-RAGE primary Ab was incubated for overnight at 4 °C. Then the membranes were washed and incubated with HRP-conjugated anti-rabbit polyclonal IgG (MP Biomedicals Inc., Solon, OH, USA) at RT for 1 h. Labeled bands were visualized using an enhanced chemiluminescence system (GE Healthcare Bio-Science, Pittsburgh, PA, USA) and exposed to high-performance chemiluminescence film (GE Healthcare). The intensity of the protein bands in Western blotting was quantified using National Institutes of Health Image 1.63 software.

### 4.9. Cell Viability Test

Cell viability was monitored after incubation with siRNA for 48 h by MTT (3-[4,5]-2,5-diphenyltetrazolium bromide) assay. Briefly, cells were harvested and incubated with MTT (0.5 mg/mL; final concentration) for 3 h. Formazan crystal was solubilized by adding dimethyl sulfoxide for 16 h. Dehydrogenase activity was expressed as absorbance at 570 and 630 nm.

### 4.10. In Vitro Scratch Assay

Cells were scratched with a sterile P200 pipette tip according to a method previously described [51]. After repeated washes to remove the resulting debris, cells were either exposed to rHMGB1 at the concentration of 50 and 100 ng/mL for 0, 12, 24, and 48 h or pretreated with RAGE siRNA. Control siRNA were used as a negative control. The wound closure in scratch assay was monitored by phase microscopy capturing images of the same field with a 20× objective at different times, as specified. Migration of cells into the cell-free space was determined by the digital image processing software “Image J” developed by NIH. In some experiments, the cells were harvested for RNA extraction.

### 4.11. Quantitative Real-Time PCR Analysis of PCNA Expression in the Gingival Epithelial Cells

After the induction of the cell damage for 48 h with or without 300 nM of RAGE siRNA, cells were collected and lysed with TRIzol Reagent according to the manufacturer’s instruction and performed with nearly the same protocol with VEGF mRNA expression analysis. The selected specific primer was purchased from Biomol International (Plymouth Meeting, PA, USA). The PCR conditions were 95 °C for 30 s, followed by 1 min annealing at 58 °C and then followed by 1 min extension at 72 °C for a total of 45 cycles.

### 4.12. Statistical Analysis

Experimental values are given as mean ± S.D. Statistical significances between different groups were assessed by one-way analysis of variance (ANOVA) test or Student’s paired *t*-test. The correlations among three examiners and the wound area measurement were calculated using Pearson’s correlation coefficient. All calculations were performed employing Sigma Stat for Windows, version 3.5 (Systat Software, Inc., Chicago, IL, USA). *p* values < 0.05 were considered statistically significant.

## Supplementary Materials

Supplementary materials can be found at www.mdpi.com/1422-0067/17/11/1961/s1.
