# Supplementary material for: HMGB1 Promotes Intraoral Palatal Wound Healing through RAGE-Dependent Mechanisms
Source: Int J Mol Sci. 2016 Nov 23;17(11):1961. doi: 10.3390/ijms17111961 (PMC5133955; doi:10.3390/ijms17111961)
Supplement: Supplementary file 1 [file ijms-17-01961-s001.pdf]

Supplementary Materials: HMGB1 Promotes Intraoral Palatal Wound Healing through RAGE-Dependent Mechanisms

Salunya Tancharoen, Satoshi Gando, Shrestha Binita, Tomoka Nagasato, Kiyoshi Kikuchi, Yuko Nawa, Pornpen Dararat, Mika Yamamoto, Somphong Narkpinit and Ikuro Maruyama

A

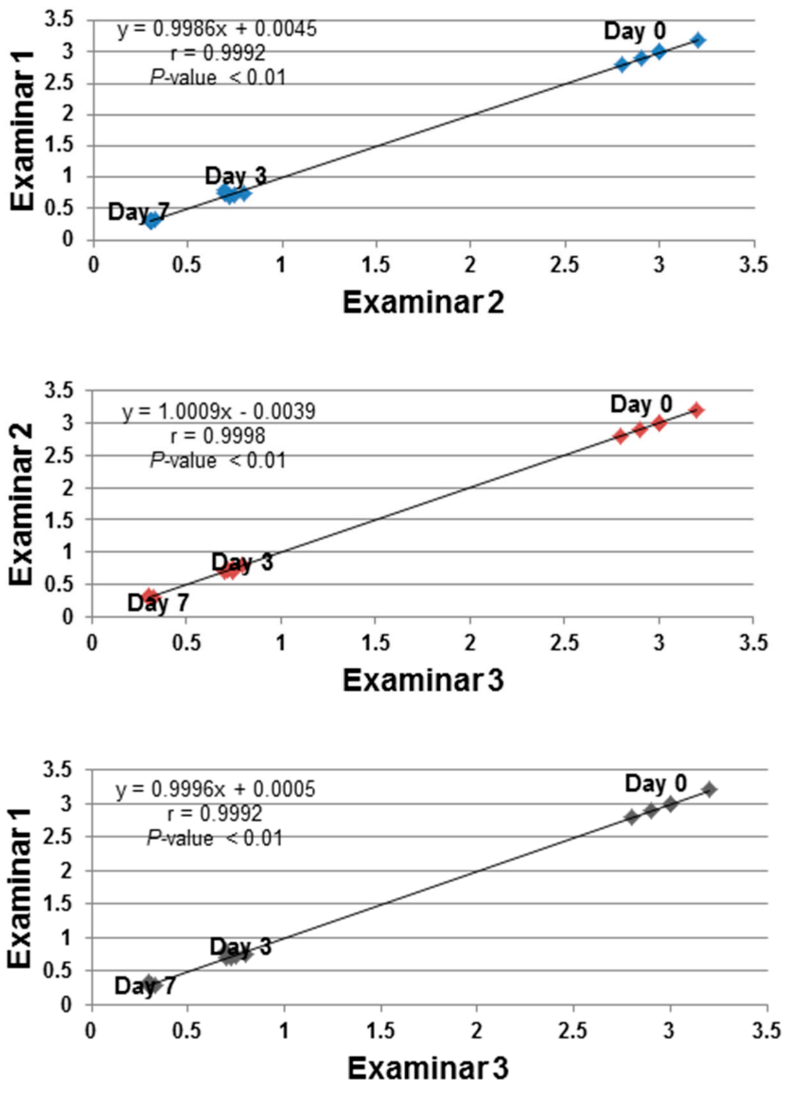

Figure S1. *Cont.*

B

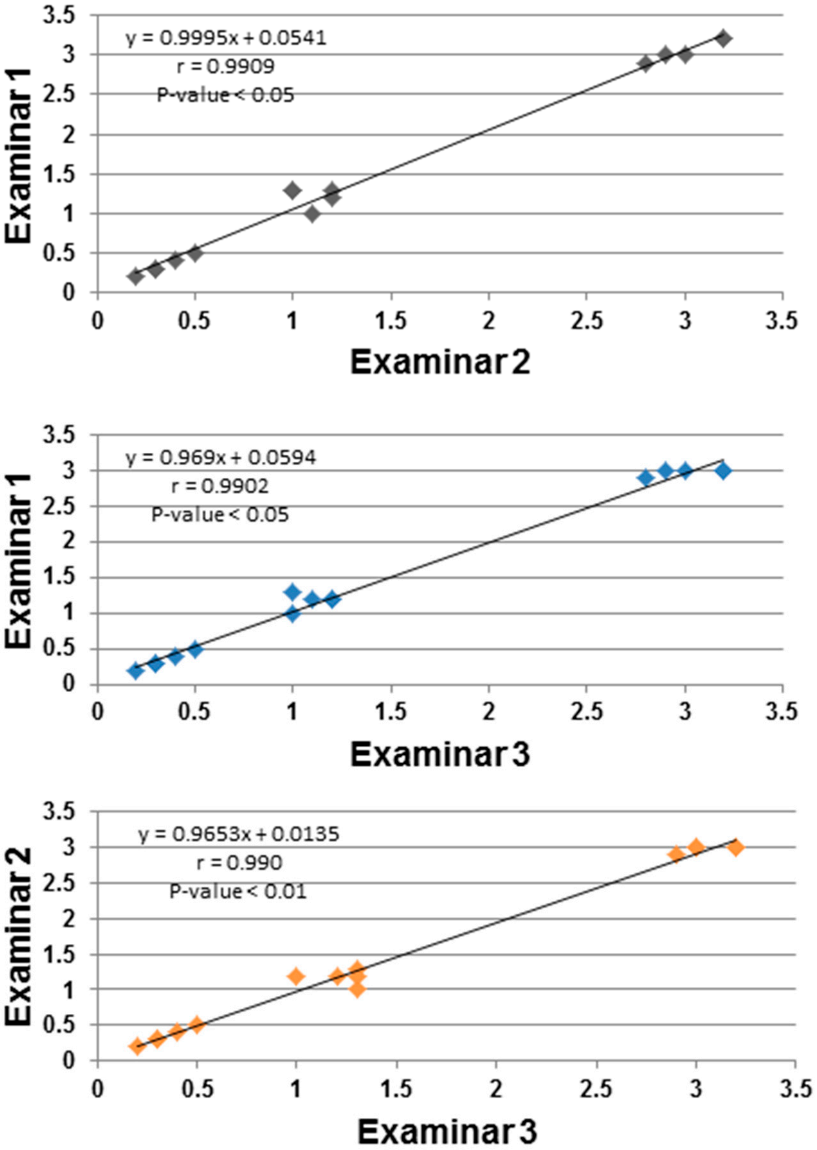

Figure S1. Inter-examiner reliability of wound area measurement. The average values of wound areas on days 0, 3, and 7 in (**A**) WT and (**B**) *Hmgb1*$^{+/-}$ mice were measured by three examiners. Pearson's correlation coefficient (*r*) values between the examiners and *p* values are shown. *p* value < 0.05 was considered statistically significant.

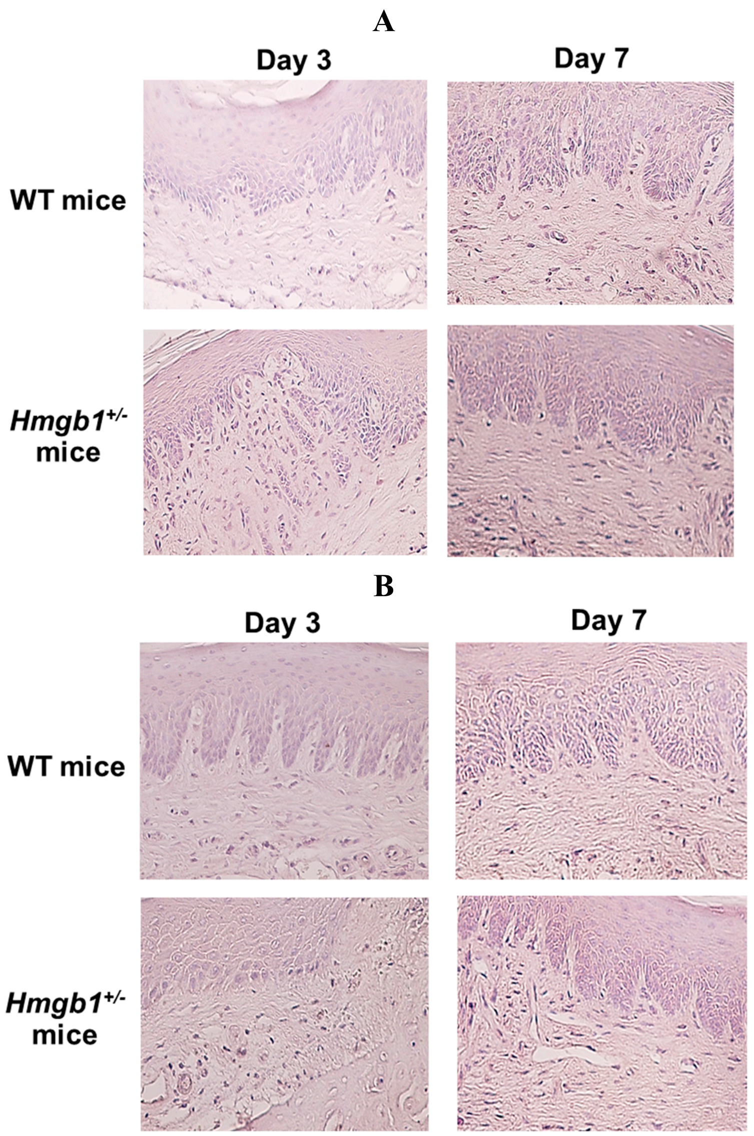

Figure S2. Analysis of antibody specificity in immunohistochemistry study. The palatal wound samples of *Hmgb1*$^{+/-}$ mice and WT mice after day 3 and day 7-post surgery were immunostained with anti-rabbit IgG at (**A**) 1:200 and (**B**) 1:500 dilution. No positive staining of the tissues in *Hmgb1*$^{+/-}$ mice or WT mice samples. All images were obtained at 400× magnification.
